# Spontaneous internal ilio-iliac fistula in an elderly woman presenting as heart failure

**DOI:** 10.2349/biij.2.2.e13

**Published:** 2006-04-01

**Authors:** GP Tan, BJJ Abdullah, S Kunanayagam

**Affiliations:** 1 Department of Biomedical Imaging (Radiology), Faculty of Medicine, University of Malaya, Kuala Lumpur, Malaysia; 2 Department of Medicine, Faculty of Medicine, University of Malaya, Kuala Lumpur, Malaysia

**Keywords:** Ilio-iliac fistula, heart failure, CT, angiography

## Abstract

Acquired intra-abdominal arteriovenous fistulas (AVFs) are a rare disorder where the communication most commonly occurs between abdominal aorta and inferior vena cava. Ilio-iliac AVF has been reported previously, but is exceedingly rare. We present a case of spontaneous ilio-iliac AVF in an elderly woman who presented with symptoms of right heart failure where the diagnosis was not considered. The computed tomographic (CT) and angiographic features are described. The current status of management as well as a review of the literature is also presented.

## CASE REPORT

An 81-year-old woman presented with palpitations, breathlessness and bilateral lower limb swelling one month prior to admission. Her symptoms had worsened five days prior to admission. She had a history of paroxysmal supraventricular tachycardia (SVT). There is no history of previous surgery, severe abdominal pain, penetrating or blunt trauma to the abdomen.

On admission, she was tachypnoeic. Her blood pressure was 120/80 mmHg. Her jugular venous pressure was raised with bilateral basal lung crepitations and bilateral lower limb pitting oedema to the level of the knees. A systolic murmur was heard at the apex of the heart. Hepatomegaly was also found. There was also a pulsatile mass per abdomen, which was clinically consistent with an abdominal aortic aneurysm.

Electrocardiogram (ECG) showed that the patient was in atrial fibrillation. Chest radiograph showed cardiomegaly with fluid in the left oblique fissure and prominent hilar vessels. A clinical diagnosis of congestive cardiac failure secondary to atrial fibrillation was made. In view of the pulsatile abdominal mass, an ultrasound examination of the abdomen was performed on the same day to assess the size of the abdominal aortic aneurysm. The right atrium, inferior vena cava and hepatic vein were enlarged along with ascites consistent with right heart failure. The abdominal aorta was ectatic and aneurysmal with the widest diameter measuring 3.7 cm. An echocardiogram, performed for further assessment of the cardiac function, showed moderate tricuspid regurgitation with enlarged right atrium and ventricle. Left ventricular function was satisfactory, with an ejection fraction of 61%.

**Figure 1:**
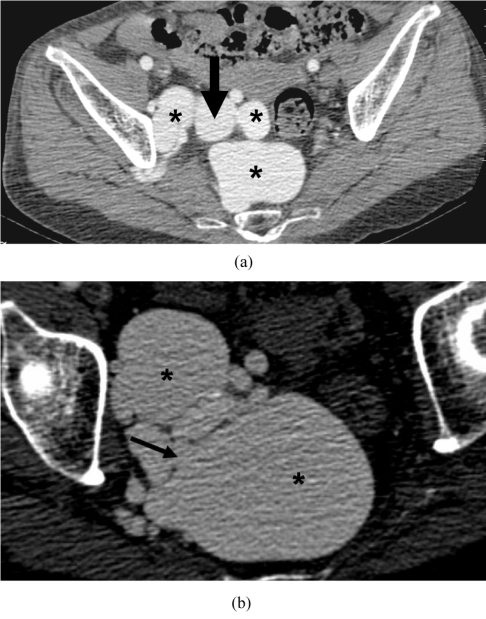
Contrast enhanced Axial CT at the level of the pelvis. (a) The enlarged tortuous right iliac artery (arrow) and right internal iliac veins (asterisk); (b) Axial image at a lower level shows the AV fistula (arrow) from aneurysmal right internal iliac artery into the internal iliac vein. Dilated veins on either side (asterisk).

**Figure 2:**
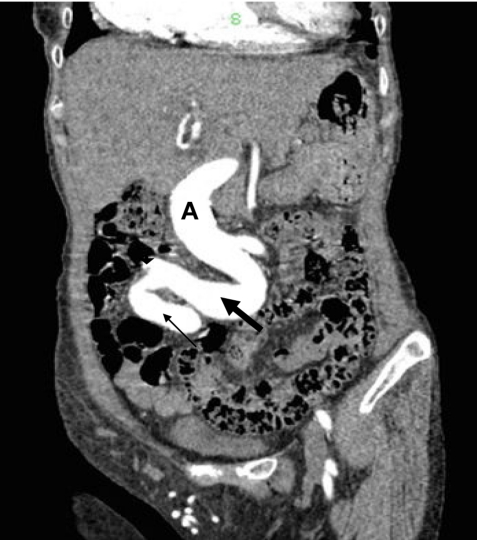
Coronal reconstruction of CT scan shows tortuous atherosclerotic aneurysmal abdominal aorta (A), right common (thick arrow) and internal iliac arteries (thin arrow) and origin of the right external iliac artery (arrowhead).

**Figure 3:**
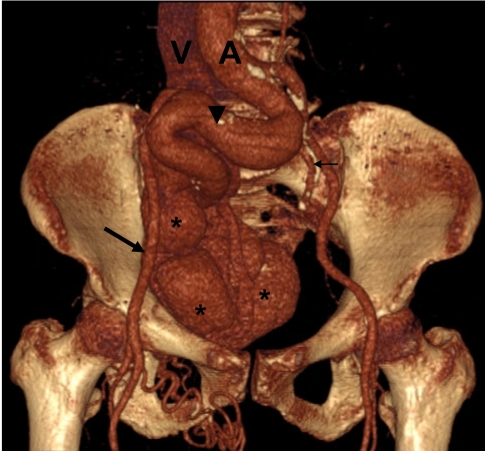
3D reconstruction of CT scan clearly shows markedly dilated right common and internal iliac artery (arrowhead) and normal right external iliac artery (arrow). The dilated tortuous right internal and common iliac veins are also noted (asterisk). The normal contralateral left internal iliac artery shown (thin arrow). A is aorta; V is inferior vena cava.

**Figure 4:**
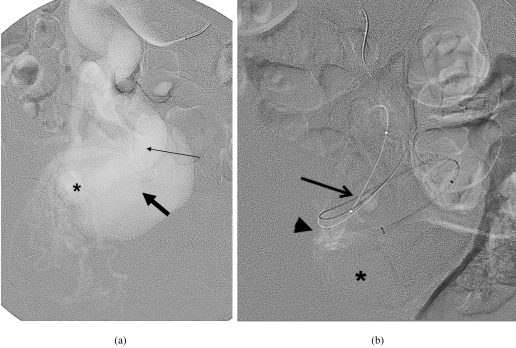
Digital subtraction angiogram. (a) Selective catheterisation of the right internal iliac artery (thin arrow) shows enlarged tortuous vessel with rapid filling of ectatic the right internal vein (thick arrow) via the fistula (asterisk); (b) Selective catheterisation of the distal right internal iliac artery with a microcatheter (thin arrow) shows large short fistulous tract (arrowhead) with some filling of ectatic the right internal vein (asterisk).

Two days after admission, CT angiogram of abdominal aorta was performed (GE High-speed, Milwaukee, WI) for further assessment of the aneurysm. The angiogram confirmed the findings of a grossly dilated right atrium and aneurysmal abdominal aorta. Furthermore, multiple markedly dilated vessels were noted in the pelvis, with a fistulous communication between the right internal iliac artery and vein (Figures 1, 2 and 3). A digital subtraction angiogram via a left branchial artery puncture of the right internal iliac artery confirmed the presence of a large fistulous communication between the right internal iliac artery and vein (Figure 4a). Both the right internal iliac artery and vein were tortuous and dilated. Superselective catheterisation of right internal iliac artery using micro-catheter (for better assessment of the location and size of the fistula) was performed as it was not possible to confirm the site of the fistula using the 4F Cobra catheter (Figure 4b). Embolisation of the fistula was not feasible due to its large size, extremely tortuous vessels, short neck, and high-flow. The patient was considered a poor candidate for surgery due to a poor premorbid status, advancing age and her reluctance and fear. Patient was discharged following treatment of heart failure.

## DISCUSSION

Arteriovenous malformations (AVMs) of the female pelvis are uncommon. These may either be congenital or acquired. Congenital pelvic AVMs are characterised by a large number of arterial feeding branches. The arterial feeders are considered undifferentiated vascular structures following arrested embryonic development at various stages [1]. Acquired pelvic AVMs on the other hand, develop following spontaneous rupture of an atherosclerotic aneurysm into the adjacent veins or following penetrating trauma (less than 20%), e.g. gunshot wound or stabbings, or after lumbar disc surgery [2]. Therefore, acquired AVMs are most commonly arteriovenous fistulas (AVF). The AVFs are most commonly aorto-caval fistula, followed by ilio-iliac [3] and aorto-iliac. However, the aetiology, clinical features, pathophysiology, principles of management and postoperative care for these fistulas are similar. Approximately 3 to 4% of all patients undergoing surgery for ruptured aorto-iliac aneurysm are found to have AVF [3]. These carry a better prognosis than intraperitoneal, retroperitoneal or enteric rupture of aorto-iliac aneurysms [5]. The disorder has a marked male preponderance [4]. Other rarer causes of acquired AVFs include Marfan’s syndrome, Ehler-Danlos syndrome, syphilis, Takayasu’s arteritis, invasion by malignant tumour [2] and in some the cause is never ascertained.

Pelvic AVMs may manifest with symptoms of pain, haemorrhage, haematuria, dyspareunia, or congestive heart failure, or symptoms secondary to mass effect on adjacent pelvic structures. Acquired AVF, secondary to surgery or trauma, tend to occur in younger patients as trauma or surgery tends to affect younger patients. In contrast, spontaneous perforation of atherosclerotic aneurysm into adjacent veins tends to occur in the older population. Two large series report mean ages of 67.3 [6] and 69.7 years. The time of onset of symptoms is usually earlier, from hours to weeks [3]. The initial diagnosis is often that of an abdominal aortic aneurysm, AVF is often not suspected and often the diagnosis is only made at surgery [5].

Iliac artery aneurysms and ilio-iliac fistulas are usually associated with abdominal aortic aneurysm, as demonstrated in this case, though they may occur as isolated entities. Rupture of atherosclerotic aneurysms into the iliac vein may have three different clinical manifestations: sudden onset of high output cardiac failure; pulsatile lower abdominal mass associated with bruit and thrill; or unilateral intermittent claudication or venous congestion. Our patient presented with the first two clinical manifestations and the tricuspid regurgitation was most likely secondary to grossly dilated ventricle from high output cardiac failure.

Ultrasound, CT or even MRI is almost always ordered for assessment of the abdominal/iliac aneurysms. Colour Doppler ultrasound has demonstrated the AV fistulas as areas of high velocity turbulent flow with aliasing of colour signal and also shows any associated thrombus at the aneurysm or fistula. Detection of ilio-iliac fistulas may however be difficult, as demonstrated by this case, as these tortuous and aneurysmal vessels lie deep within in the pelvis and obscured by overlying bowel gas. CT angiography is excellent in demonstrating the aneurysm and fistulous communications [6], especially with the advent of multi-detector CT and 3D software. Early contrast opacification of the iliac veins and inferior vena cava, and site of the fistula are clearly visualised.

Conventional angiography still remains the ‘gold standard’ for assessment of AVMs as well as assisting in assessing options for endovascular management. Endovascular treatment has gained considerable favour in the management of arteriovenous fistula especially for those with significant high-risk comorbid factors. Options available include percutaneous endovascular treatment with covered self-expanding stent graft to cover the mouth of the fistula. However, this is not always feasible due to the tortuous iliac vessels. Transcatheter embolisation with coils or detachable balloons [7] is generally not recommended due to the large size of the fistula, high flow and short neck. Surgical options for AVF consist of endo-aneurysmal repair of the fistula and prosthetic graft replacement of the aortoiliac aneurysm but these are associated with high morbidity and mortality (approaching 60%) due to the emergent nature of the procedure [4]. Thus early diagnosis and appropriate management is of paramount importance.

Unlike acquired AVFs, surgical treatment of congenital AVMs is difficult due to the extensive nature and the large number of dysplastic feeder vessels with the potential for exsanguinating haemorrhage and damage to surrounding structures [8]. Accordingly, transcatheter embolisation has become the treatment of choice [8]. Preoperative embolisation of AVMs has also been used as an adjunct to decrease intraoperative blood loss. Small asymptomatic AVMs that do not increase in size may be safely observed.

AVFs between major abdominal vessels are uncommon complication of aortoiliac aneurysm. Ilio-iliac fistula in a female patient is even rarer and associated with high morbidity and mortality especially if the diagnosis is not suspected. In this patient, in the absence of history of trauma or surgery and the presence of extensive aneurysmal disease involving the aorta and iliac arteries, it is reasonable to believe that the AV fistula was secondary to perforation of the aneurysmal right iliac artery into the right iliac vein. In addition to the rapid onset, the presence of a single communication and older age group lend more support to this diagnosis. There has only been a single reported case in the literature with an ilio-ilial fistula secondary to atherosclerotic disease [5]. In addition the CT angiographic appearances of ilio-ilial AVF have not been described.

## References

[1] Diwan RV, Brennan JN, Selim MA (1983). Sonographic diagnosis of arteriovenous malformation of the uterus and pelvis. JCU.

[2] Farid A, Sullivan TM (1996). Aortocaval fistula in a ruptured inflammatory abdominal aortic aneurysm. J Cardiovasc Surg..

[3] Kazmier FJ, Harrison CE (1973). Acquired aortocaval fistulas. Am J Med..

[4] Ghilardi G, Scorza R, Bortolani E (1993). Rupture of abdominal aortic aneurysms into the major abdominal veins. J Cardiovasc Surg..

[5] Davis PM, Gloviczki P, Cherry KJ (1998). Aorto-caval and ilio-iliac arteriovenous fistulae. Am J Surg.

[6] Adili F, Balzer JO, Ritter R-G (2004). Ruptured abdominal aortic aneurysm with aorto-caval fistula.. Journal of Vascular Surgery.

[7] Saim Y, Abdullah E, Ersin L (2004). Transvenous embolisation and stent placement for an internal iliac arteriovenous fistula with central iliac vein occlusion. Journal of Vascular and Interventional Radiology.

[8] Jacobowitz GR, Rosen RJ, Rockman CB (2001). Transcatheter embolization of complex pelvic vascular malformation: results and long-term follow-up. J Vasc Surg.

